# A qualitative study of young peoples’ thoughts and attitudes to follow a more plant-based diet

**DOI:** 10.3389/fpsyg.2023.1196142

**Published:** 2023-08-31

**Authors:** Catherine McInnes, Sharon A. Carstairs, Joanne E. Cecil

**Affiliations:** ^1^School of Medicine, University of St Andrews, St Andrews, Fife, Scotland, United Kingdom; ^2^School of Health Sciences, University of Dundee, Dundee, Scotland, United Kingdom

**Keywords:** plant-based diets, young people, theory of planned behavior, intentions, attitudes, qualitative, education

## Abstract

Plant-based diets (PBDs) refer to dietary habits that reduce the consumption of animal-based products and increase the consumption of nutritionally rich plant foods. PBD’s have been shown to provide significant health benefits, such as reducing obesity and improving psychological wellbeing, and are environmentally friendly. However, few studies have investigated factors that influence young people’s thoughts and attitudes toward following a PBD in western societies, particularly in the United Kingdom. Understanding these factors may benefit public health interventions that encourage the consumption of more fruit and vegetables. The aim of this study was to explore the factors that affect young people’s intentions toward following a PBD. Twenty-one young people (18–24 years) participated in this qualitative study. Participants were asked about their views of PBDs in a semi-structured interview. Thematic analysis was utilized to explore views and the barriers and facilitators to following a PBD. The Theory of Planned Behavior (TPB) was used as a framework to organise the findings. Within attitudes, the sub-themes identified were an awareness of a healthy diet, environmental concerns, health concerns and distrust, perceptions of PBDs and associated stereotypes, perceived restriction and lack of enjoyment, and need for education. Within subjective norms, the sub-themes identified were cultural and familial norms, peer influence, and exposure through social media. Within perceived behavioral control (PBC), the sub-themes identified were a lack of independence and parental control, lack of knowledge and perceived difficulty, lack of inclusiveness and accessibility, and inconvenience. Overall, the findings suggest that increased provision of education and knowledge about PBDs to young people, and widening access to PBDs, could encourage and help improve their understanding and intention to follow this dietary style. Tailored health promotion strategies, which also consider additional barriers and facilitators found within this study, could motivate young people to consume a more PBD.

## Introduction

The consumption of animal-based food products has been rising globally, for example, since the 1960’s the production of meat-based products as part of the global food supply has increased by 204% (Basu, 2015). This rise in meat consumption is particularly evident within high-income countries, such as the United Kingdom (Stewart et al., 2021). According to Riley (2010), meat has remained a key part to a traditional British diet and is a fundamental component of people’s cultural identity. However, diets that contain high amounts of animal-based food items have been associated with negative health outcomes, namely colorectal cancer (Bouvard et al., 2016) and obesity (You and Henneberg, 2016), as well as being environmentally unsustainable (Machovina et al., 2015). Therefore, it is now accepted that shifting toward a plant-based diet (PBD) may help to improve health and wellbeing (Krizanova et al., 2021), and reduce the negative effects of meat consumption on the environment (Machovina et al., 2015).

PBD’s represent dietary habits that reduce the consumption of animal-based products, namely meat, and increase the consumption of nutritionally rich plant foods, including vegetables, fruits, and wholegrains (Kent et al., 2022). These plant-based foods are rich in fibre and low in saturated fat and have been demonstrated to benefit physical health through reducing the likelihood of developing Type II diabetes, cardiovascular disease (Sabaté and Wien, 2010; Hemler and Hu, 2019) and obesity (Huang et al., 2016). Some plant-based foods are nutritionally incomplete, such as meat substitute products, which have been shown to contain fewer vitamins, for example, B_12_, and high levels of salt (Penna Franca et al., 2022). Recent evidence has also shown that diets high in nutritious plant foods can reduce adult’s risk of developing colorectal cancer (Kim et al., 2022). As well as improving physical health, reducing meat intake can improve people’s mood (Lee et al., 2021) and cognitive functioning (Zhu et al., 2022). Together, these data suggest that PBD’s may help to benefit physical and mental health in young people.

The negative consequences of animal-based food consumption are also being debated from an environmental perspective (Kahiluoto et al., 2014; Raphaely and Marinova, 2014), as evidence suggests that meat production produces 48% more greenhouse gases (GHG) into earth’s atmosphere compared with plant-based food production (Werner et al., 2014). These apprehensions have called for greater awareness to be raised about transitioning toward a PBD based, and education to be provided on the benefits provided to young people’s health and the environment (Kahiluoto et al., 2014). Environmental concerns from eating meat can be a key motivator for people to adopt vegan and vegetarian diets (Mullee et al., 2017; Schenk et al., 2018). However, few studies have analysed whether environmental concerns may influence young people’s intentions toward adopting a PBD.

Although there are benefits to shifting toward PBD’s, several barriers exist that may hinder people in western societies from adopting this dietary style (Lentz et al., 2018). These barriers include the predominant role that animal-based products have in western diets due to their associations with power and affluence (Von Essen, 2021). Cooking meat is also viewed as a traditional practice within western societies, as the behavior of eating meat has been consolidated into their understanding of nutrition and meal preparation (Hoek et al., 2017). Therefore, efforts to reduce meat consumption should take into consideration systematic and personal barriers to dietary change (Lentz et al., 2018). Understanding these barriers can inform the development of interventions to help reduce meat intake and promote a healthy, balanced diet within western societies (Hoek et al., 2017).

Young people, defined to be aged between 10 and 24 years (WHO, 2021), have been identified as an age group at risk of adopting unhealthy diets that contain few nutritious plant-based foods (Ensaff et al., 2015). This may be attributable to a developmental stage in attaining autonomy from primary caretakers and control over food intake (Moreno et al., 2014) and life transitions that involve a change of environment, for example when students move from home to university (Sprake et al., 2018). Consequently, unhealthy dietary decisions can exacerbate young people’s probability of developing health complications, for example obesity and diabetes (Poobalan et al., 2014; Lascar et al., 2018). Unhealthy dietary choices adopted during this developmental stage can persist into adulthood, resulting in long-term health consequences (Haines et al., 2019). Research has demonstrated that young people can be more receptive to adopt alternative trends in dietary preferences and change their behavior (Janda and Trocchia, 2001). This may be due to young peoples’ identities evolving under the influence of their environment (Krizanova et al., 2021). Therefore, encouraging young people to adopt a PBD can promote healthier dietary choices which may continue into adulthood and improve wellbeing (Ensaff et al., 2015).

Despite the recognition of the benefits of PBD’s, few studies have directly investigated young peoples’ attitudes toward PBD’s in the United Kingdom, where meat-based diets are more popular than in non-western countries (Stoll-Kleemann and Schmidt, 2017). Qualitative research by Ensaff et al. (2015) using a grounded theory approach found that British young adolescents were influenced by several psychosocial factors, such as appearance, convenience, and perceived lack of taste in PBD’s. Research conducted in the Netherlands by Havermans et al. (2021) employed the theoretical framework Reasoned Action Approach to understand intentions and behavior toward PBDs and showed that Dutch adolescents had little knowledge of PBD’s, and intentions were affected by perceived social norms and taste of PBD’s. Therefore, taste, convenience, and social norms may be influential factors in young people’s dietary choices (Daly et al., 2022). More recently, the Theory of Planned Behavior (TPB) has been used in quantitative studies to investigate attitudes toward reduction in meat consumption in adults in the United Kingdom (Coker and van der Linden, 2022) and Switzerland (Krispenz and Bertrams, 2020). Research investigating dietary behaviors has demonstrated the TPB’s components to be strong predictors of dietary change (Maki and Rothman, 2017). Further, this theoretical framework can provide a rich understanding of dietary intentions and behaviors (Rosenfeld and Burrow, 2018).

The aim of this study was to investigate the factors that may influence young people’s views and intentions to adopt a PBD, using the TPB as a framework for organising the findings. Additionally, this study aimed to identify the barriers and facilitators that affect young people adopting a more PBD. Understanding these factors may help to guide the formation of interventions that encourage young people in the United Kingdom to adopt more PBD’s, which have considerable benefits for health and wellbeing (Kent et al., 2022).

## Methods

### Design

This study utilized a within-subjects qualitative research design.

### Participants and recruitment

Participants were required to be of British nationality, living in the UK, and aged between 18 and 24 years, following the WHO (2021) definition of young people. Eligible participants were those not currently consuming a vegan, vegetarian, or PBD. Young people who identified as vegan and vegetarian were not recruited as this study focused on identifying the attitudes of young people potentially transitioning to a more PBD. Participants were recruited through convenience sampling using online advertisements posted on Facebook. A target sample size of 10–24 participants was determined based on recommendations from the literature (Malterud et al., 2016; Hennink et al., 2017) to capture the depth and insights of the studied phenomenon, ensuring content validity (Hennink and Kaiser, 2022). Participants did not receive credit for participation, but did receive a £10 Amazon voucher as recompense.

### Procedure and materials

Ethical approval was granted by University of St. Andrews Medicine Ethics committee (MD16132). Consenting participants took part in individual interviews conducted between May and June 2022 via Microsoft teams. Interviews were audio recorded using a mobile app called Voice Memo’s, and semi-structured to allow for an unobtrusive insight into participants experiences, while allowing the researcher to keep the discussion focused (McIntosh and Morse, 2015). There was no time limit set for conducting interviews, as interviews finished when all questions had been asked. Interview lengths ranged from 14 to 45 min.

The interview schedule was developed and informed using the TPB, similar to previous research (Zoellner et al., 2012). The schedule was edited after being pilot tested, following Bearman’s (2019) recommendations for finalizing an interview schedule. The final interview schedule consisted of 27 questions (Supplementary material), including demographic questions (age, gender, ethnicity, level of education) and were primarily open-ended to allow for in-depth discussions on people’s attitudes, subjective norms, and PBC surrounding PBD’s.

### Analysis

The interviews were transcribed verbatim by the researcher (CM) and anonymised to protect participant confidentiality. Although no software was used to support data management or transcriptions, audio recordings were listened to multiple times and data were manually coded by the researchers.

To understand the factors that affect young people’s intentions to adopt a more PBD, the data were analysed using inductive and deductive approaches following a similar structure to previous research (Zoellner et al., 2012). The researcher also chose a critical realist perspective, which assumes that data does not fully reflect an individual’s reality and requires interpretation to identify the underlying social constructs of the data (Wiltshire and Ronkainen, 2021). The exploration of emergent sub-themes within the data was carried out using an inductive thematic analysis approach guided by Braun and Clarke’s (2006) thematic analysis procedure. The researcher (CM) re-read the transcripts multiple times to become familiarized with the data and to make notes of recurring concepts and ideas. The data were then coded initially at a latent level, by manually highlighting extracts and developing initial emergent codes. Codes were analysed and combined to develop potential sub-themes. A random sample of transcripts (20%) were reviewed for emergent codes by two researchers (JC and SC) and discussion to reach consensus was conducted. The sub-themes were then reviewed by all researchers to confirm that they reflected the coded extracts. We then utilized Ajzen’s (1985) TPB components for organising the data, reflecting a deductive approach. By organising emergent themes into the TPB components, we gain a better understanding of attitudes, behavior, and intentions (Rosenfeld and Burrow, 2018). Thus, the sub-themes were mapped onto the components of the TPB. Finally, data were presented within the results. Once data saturation had occurred, whereby no new emergent sub-themes were identified (Guest et al., 2006) by the researcher (CM), no more participants were recruited.

## Results

### Participant demographics

A total of 21 young people were interviewed. Participants consisted of 16 females and 5 males, with a mean age of 21.9 years (± 1.3). Most participants were educated to an undergraduate university level (66%), while other participants were educated to a master’s level (24%), National diploma level (5%), and Scottish higher level (5%). Participants largely identified as white British (76%), while others identified as white Scottish (14%), Chinese British (5%), and mixed British (5%). Mean length of interviews were 27.6 min (± 8.7).

### Qualitative results

**Figure 1:**
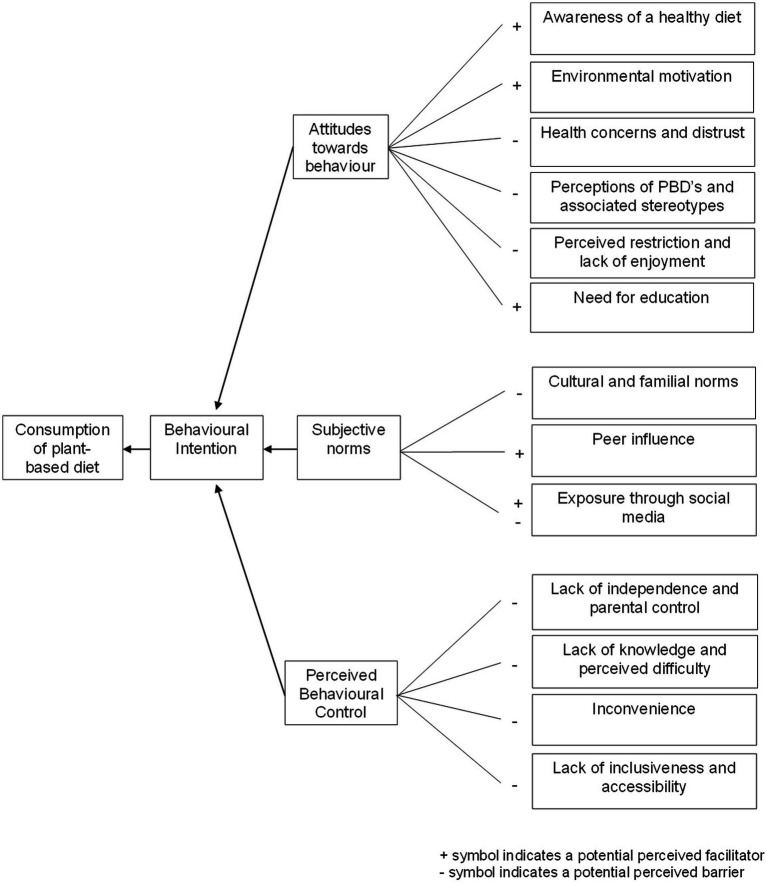
Emerging sub-themes mapped onto the Theory of Planned Behaviour components explaining the attitudes, subjective norms, and perceived behavioural control influencing young people’s intention to follow a more PBD.

The emergent sub-themes identifying factors influencing young people’s intention to adopt a more PBD were mapped onto the components of the TPB and are described below and presented in Figure 1. Within attitudes, the subthemes identified were an awareness of a healthy diet, environmental concerns, health concerns and distrust, perceptions of PBDs and associated stereotypes, perceived restriction and lack of enjoyment, and need for education. Within subjective norms, sub-themes identified were cultural and familial norms, peer influence, and exposure through social media. Within perceived behavioral control (PBC), the sub-themes identified were a lack of independence and parental control, lack of knowledge and perceived difficulty, lack of inclusiveness and accessibility, and inconvenience.

#### Attitudes

Discussions regarding the participant’s attitudes toward PBD’s revealed six main sub-themes: awareness of a healthy diet, environmental concerns, health concerns and distrust, perceptions of PBD’s and associated stereotypes, perceived restriction and lack of enjoyment, and need for education (Table 1).

**Table 1:** Sub-themes and Quotations Within the attitudes component of the Theory of Planned Behaviour.

Sub-theme	Quotation number	Quotations
Awareness of a healthy diet	1	“It is important to have a well-balanced diet because the days that I do not eat well I do feel rubbish about myself, and ill kind of go over what I’m eating and go like ‘oh I’ve not had any fruit or vegetables’” (1,004, female)
	2	“If people are incorporating more plants into their diet then that’s really amazing for their health…I notice a huge change in my mood and energy levels when I do/do not eat in those balanced proportions…I do not function well, and I get anxiety and my mood changes” (1,007, female)
	3	“I’m trying to make a conscious effort of being healthier, em, and yeah just getting fitter and healthier so I think yeah, having a well-balanced diet is key to that” (1,009, female)
	4	“When I eat better I genuinely do feel better” (1,015, female)
	5	“What you eat is basically fuel for your body, and if you eat like really unhealthy things, so like snack a lot, em, for me it tends to affect my mood and how much energy I have, so I think it is quite important for those reasons to have a well-balanced diet” (1,016, female)
Environmental motivations	1	“I think humans and environmental health really goes hand in hand, em, so I see a huge benefit in that” (1,007, female)
	2	“To like reduce meat consumption in general is a good idea, em, I think it is important because it is obviously one of the biggest contributors to greenhouse gases” (1,019, male)
	3	“I think I am motivated obviously by the, like, climate situation and climate emergency at the moment, em, but I am bad myself for just sticking to what I, not necessarily I know because I do, and I have eaten many other alternatives” (1,005, female)
	4	“I’ve seen a lot of stats about how it’s a lot better for the environment to have. Plant-based diet” (1,001, female)
	5	“I also feel quite passionately about its benefits for the planet as well as I think the statistic is that we can reduce our carbon footprint by 73% by going a bit more plant-based” (1,014, female)
Health concerns and distrust	1	“I’ve been hearing of athletes switching to plant-based diets and well, the biggest argument I hear from people against vegan or plant-based diets in general is that it’s not healthy, your lacking certain nutrients” (1,018, male)
	2	“Something I really struggle with in the plant-based world is it just feels like people are spewing out, you know, nutritional info and stats left and right, and I am not convinced that they have a deep understanding of what they are talking about” (1,007, female)
	3	“I’ve heard a lot of cases of taking meat out of a diet and then there’s been health issues associated cause obviously having a well-balanced diet and everything…I would be worried about taking any food group out of my diet without knowing what I need to do in order to replace that so I can still have a healthy diet” (1,009, female)
	4	“I’m weary of plant-based diets because I think there is a lot of disordered eating that the line is very thin there and I think I see a lot of fellow women engaging in plant-based diets and I think sometimes disordered eating is hiding” (1,007, female)
	5	“My dad…he’s always the one telling me to eat less meat…but right now I feel like my goals are different to his, he is thinking more about like longevity and health, where in a way I am thinking more of like performance and like my sports goals that requires eating meat” (1,003, male)
Perception of PBD’s and associated stereotypes	1	“My current understanding of the term is that it’s really just another way of saying vegan” (1,018, male)
	2	“People say it’s just rabbit food and it’s not going to fuel you” (1,013, female)
	3	“The classic stereotype of like a hippie with like long hair or, like does not shave for example, like I think that in some people’s eyes is the stereotype of somebody who follows a plant-based diet” (1,004, female)
	4	“It’s very much ‘you are a tree hugger, or you are a supermodel to get something’, and it’s not just doing it, you know, because you enjoy it” (1,010, female)
	5	“A lot of people have bad stigmas about plant-based diets, like’ oh it’s vegans and vegetarians’” (1,010, female)
Perceived restriction and lack of enjoyment	1	“It just makes me think ew, nah, that’s not for me and I think I would be restricting the types of things I enjoy eating so I decided against it” (1,013, female)
	2	“I’ve not ever really thought of reducing my meat intake because I do really enjoy burgers and curry’s and everything, so yeah, I’ve not really reduced any meat…mainly because I do not want to limit a whole bunch of different options” (1,009, female)
	3	“I also think that you do not want to limit yourself cause there is so much nice food out there to try” (1,006, female)
	4	“Some of the things you can get to fit a plant-based diet does not really taste as good, so I think that is one of the main reasons why I do not like or like I’m not really inclined to switch fully to a plant-based diet” (1,016, female)
	5	“I feel like my dad is punishing himself by following a plant-based diet because I do not think he truly wants to follow one” (1,004, female)
Need for education	1	“At secondary school it’s very much 5 a day and the protein side comes from meat and you never had much else” (1,012, female)
	2	“I definitely think that teaching in schools has a massive impact as it maybe is not like fully understood…I suppose facilitators would be for like government level things that I would think to make plant-based eating and the benefit of it taught about in schools” (1,014, female)
	3	“I think education has a huge part to play as there is a huge stigma around plant-based diets, but it’s also the way that they are done as well, you know, it’s not all peachy” (1,010, female)
	4	“If kids are at a young age and their getting used to seeing things that have non-meat in them…So I definitely think that there should be more advertising and education for more families and children” (1,013, female)

Awareness of a healthy diet: participants expressed a strong awareness and value of the importance of consuming a well-balanced diet, particularly through incorporating more fruit and vegetables into their diet (Table 1, Q1). This awareness encouraged the participants to view incorporating more plant-based items into their diet as beneficial for their health and wellbeing (Table 1, Q2).

Environmental motivations: the participants positive attitude toward consuming more fruit and vegetables was also expressed through environmental concerns for the planet. In particular, the participants discussed the benefits to the environment, such as a reduced impact on greenhouse gases and carbon footprint, as a result of potentially reducing their consumption of animal-based food items (Table 1, Q1, Q2).

Health concerns and distrust: although participants held positive attitudes toward PBD’s through environmental concerns and plant-based food consumption, most participants discussed concerns toward people’s health through reducing or omitting food groups which contain animal-based foods (Table 1, Q1). This concern was expressed through a distrust toward the benefits of consuming PBD’s (Table 1, Q2), which led most participants to believe that PBD’s may reduce their likelihood of achieving a well-balanced diet (Table 1, Q3). Additionally, it was observed that health concerns were expressed differently between males and females, with only females expressing concerns toward developing disordered eating while predominantly males expressed concerns toward restricting protein that they believed was needed for physical performance and strength (Table 1, Q4, Q5).

Perception of PBD’s and associated stereotypes: the participants’ reported confusion about vegan diets (Table 1, Q1), holding negative attitudes and attaching negative stereotypes of vegan diets to PBD’s (Table 1, Q2, Q3). More specifically, the participants stigmatized their intentions to follow PBD’s through their negative associations of veganism with PBD’s (Table 1, Q4, Q5).

Perceived restriction and lack of enjoyment: the association of vegan diets to PBD’s caused participants to perceive PBD’s as being restrictive (Table 1, Q1, Q2). More specifically, PBD’s were expressed as restricting the food options available to the participants (Table 1, Q3). This perceived restriction led participants to believe that PBD’s were not enjoyable and may prevent participants from consuming non-plant-based foods that were considered pleasurable to eat (Table 1, Q4, Q5).

Need for education: the participants described the lack of education they have received on PBD’s (Table 1, Q1). This lack of education, particularly from a young age, meant that there is often an uncertainty about PBD and of the benefits of consuming a more PBD (Table 1, Q2). Additionally, participants expressed that a lack of education on PBD’s may be further contributing to the stigmatization of PBD’s (Table 1, Q3). Therefore, participants expressed a greater need for education to be given on PBD’s from a young age (Table 1, Q4), to help bring better awareness of PBD’s.

#### Subjective norms

Discussions regarding the perceived influence of subjective norms toward participants diet and following a PBD elicited three main sub-themes: cultural and familial norms, peer influence, and exposure through social media (Table 2).

**Table 2:** Sub-themes and quotations within the subjective norms component of the Theory of Planned Behaviour.

Sub-theme	Quotation number	Quotations
Cultural and	1	“A lot of our main traditional dishes are, you know, curry or haggis that are all meat-based, you know, like why do not we have like a national dish that is not like that…even if you wanted to become plant-based meat is shoved in your face” (1,010, female)
familial norms	2	“One of my friends who is vegan has a partner and that he has now become vegan, but he has to like, when he goes home at Christmas time his family will not allow him to be vegan for Christmas dinner so I think this would be kind of similar with my family as well” (1,009, female)
	3	“I’m from quite a rural area in Scotland, so we are all brought up to eat meat and eggs and stuff…when you have been brought up on a meat-based diet, you kind of just want to keep eating it, um, and I’m a bit scared of trying stuff new, new stuff” (1,008, female)
	4	“I think older people kind of look down on younger people that are plant-based, em, being a little bit maybe judgy and thinks we are a little bit like alternative and hippie and stuff” (1,006, female)
	5	“Maybe my family could be a bit judgy if I tried, if I was to try and not, em, try and eat a plant-based diet, especially when I go home and my parents do a lot of the shopping and the cooking and they’d be like ‘ugh that’s so annoying’” (1,001, female)
Peer influence	1	“My flatmate…when we used to cook together, em, I used to like having the vegetarian option cause she used to give it to me…and then I could try it without having to commit to buying it so it was really definitely nice” (1,012, female)
	2	“We’ve made veggie lasagne, we you know, seeing what she makes and smelling it and going ‘mmm, that’s actually really nice” (1,010, female)
	3	“One of my friends who is…I think she is plant based, I always used to have soya milk because that’s always what the doctor used to recommend when I was allergic years and years ago, but she kind of told me about oat milk and some alternative products, and I tried them and I have never gone back now” (1,009, female)
	4	“Both my flatmates, well one was veggie and one was attempting to go vegan…when we lived in the flat together it was definitely something that, you know, we’d cook meals together that would always be plant-based because then we could all eat it” (1,005, female)
	5	“When you see other people living a certain way it does kinda make you think like ‘oh maybe I could do that’ or, and like we sometimes when we go out to eat we will choose places that are more, that cater more for like people who have a plant-based diet” (1,006, female)
Exposure through social media	1	“Seeing social media influencers and like different wellness influencers, like they tend to follow quite a plant-based diet and seeing different products makes me more inclined to want to try it and adopt that kind of diet” (1,016, female)
	2	“I look on Beyond meat more favorably since Kim Kardashian likes it” (1,014, female)
	3	“I think that on social media as well, you know, if something looks good and it’s part of a plant-based diet then I will want to eat it” (1,013, female)
	4	“With plant-based diets becoming quite a trend and trending on socials and stuff like that, so I think obviously, there is obviously the risk then of people adopting that without knowing how to adopt it healthily” (1,009, female)
	5	“I think it is the craze at the moment in terms of diets, em, like I follow deliciously Ella, I followed her for ages, like I love her videos and I like her recipes” (1,004, female)

Cultural and familial norms: most participants expressed that following a PBD was not in line with their perceived cultural and familial norms surrounding meat consumption (Table 2, Q1, Q2, Q3) and often resulted in the avoidance of adopting a PBD’s due to these negative opinions of family members, particularly from older family members (Table 2, Q4, Q5).

Peer influence: despite the cultural and familial norms surrounding meat consumption, participants expressed that PBD’s were more normative and acceptable if they knew peers who consumed vegan, vegetarian, or PBD’s (Table 2, Q4). Having these peers allowed participants to experience new foods and better understand PBD’s (Table 2, Q5).

Exposure through social media: the participants also expressed that exposure to dietary information through social media allowed them to view PBD’s as normative behavior (Table 2, Q3). This exposure allowed the participants to learn new recipes, particularly when promoted by influential celebrities. However, some participants expressed that viewing and being influenced by PBD information online can be negative due to their perceived ‘trendiness’, and individuals may receive incorrect information to adopt a healthy PBD (Table 2, Q4).

#### Perceived behavioral control

Discussions regarding the participants PBC in following a PBD revealed four sub-themes: lack of independence and parental control, lack of knowledge and perceived difficulty, inconvenience, and lack of inclusiveness and accessibility (Table 3).

**Table 3:** Sub-themes and quotations within the perceived behavioral control component of the Theory of Planned Behaviour.

Sub-theme	Quotation number	Quotations
Lack of independence and parental control	1	“With my mum telling me like ‘oh do not eat veggie X, Y, Z’, em, at first it wasn’t that I would not do it, it was just in the back of your head it would be like ‘oh well I do not need to’ because you need to listen to what your mum says” (1,019, male)
	2	“Still living from home and not being fully in control of what you eat” (1,014, female)
	3	“I’m trying to encourage it in my family but it’s a balancing act that is not working too well” (1,012, female)
	4	“If I go home then I guess it’s like you go back into patterns of what the family buys…if you still live with your family and other people and what they cook around you, say if you are like cooking together and you want something different then it can be quite a difficult thing” (1,000, female)
	5	“It’s hard cause I live at home, I think when I live, like when I move out then that will be easier cause obviously, I’ll control what food is bought and stuff” (1,006, female)
Lack of knowledge and perceived difficulty	1	“I guess defining what and how much fruit and veg actually make a diet plant-based is part of the challenge” (1,017, male)
	2	“Sometimes you just want a kebab after a night out and you have to know the plant-based alternatives for that” (1,016, female)
	3	“I still describe it easier to cook with meat rather than plants though as in my mind I’m like ‘oh just throw meat in it and its finished’ you know” (1,019, male)
	4	“A lot of people know how to cook meat quite easily, whereas vegetables you kind of have to think ‘oh, what do I have to do to cook a veggie lasagne’ or you have to kind of go out of your way a little bit” (1,010, female)
	5	“I could cook with it but I just, and I do not know if I would be able to make it taste that great, like from my imagination I think it would be harder because, yeah, like I do not even know what I would cook” (1,004, female)
Inconvenience	1	“I can be picky with certain foods, I find it hard to commit to eating a plant-based diet and I find that convenience wise it’s a lot easier to not do that” (1,013, female)
	2	“You’re still looking at two or three options on a menu and it’s still mostly meat-based options, and sometimes if you want to go vegan you have to look for specific restaurants” (1,010, female)
	3	“I think being a student does add to it, and then like you know when I go into a 9 to 5 job I’m still gonna probably have more time as a student so it’s like I do not know, I feel like you, you know you are always busy, you are always going to have other things to do and you take time out within your day to cook a big meal I do not know?” (1,004, female)
	4	“I would not go out of my way to find somewhere that was more plant-based, whereas if it became more mainstream to offer these things then I think that’s a good thing” (1,001, female)
	5	“It does require higher transition to buying different kinds of things and like be more experimental in your cooking, em, which can be a barrier if people do not have the time, or yeah” (1,000, female)
Lack of inclusiveness and accessibility	1	“For the foreseeable future a plant-based diet is going to be a primarily middle class and above thing, it’s not going to be something that a lot of people will be adopting simply because they cannot afford it” (1,018, male)
	2	“To commit to that sort of diet I think in my experience is quite a middle-class thing to do…now that people are going to food banks to get whatever food they can, to be able to say ‘oh no I only eat vegan’ is such a luxury” (1,014, female)
	3	“A lot of the unhealthy foods are really cheap like your bag of crisps, like why on earth is McDonalds cheeseburgers 99p, and then a salad is your looking at four or five quid, and that’s the imbalance of it” (1,010, female)
	4	“I can see more middle to higher income people adopting more of a plant-based diet as I cannot see it being something that lower income people or people struggling financially would adopt” (1,009, female)
	5	“It’s not always accessible for everyone in terms of, like, sometimes it can be more expensive” (1,006, female)

Lack of independence and parental control: a lack of independence and parental control over the participants dietary choices appeared to influence participant’s perceived behavioral control in following a PBD (Table 3, Q1). Due to perceived complexities around family consumption behavior (Table 3, Q4), participants indicated that they would be motivated to adopt a PBD when they move out of their family home and have greater autonomy over their dietary choices (Table 3, Q5).

Lack of knowledge and perceived difficulty: the participants discussed the lack of knowledge they have toward PBD’s, particularly in understanding the composition of PBD’s and the alternative food options available (Table 3, Q1, Q2). This lack of knowledge encouraged participants to view PBD’s as difficult to engage in, as meat was regarded as easier to prepare and tastes good compared with vegetables (Table 3, Q3, Q5).

Inconvenience: most participants discussed the perceived inconvenience toward following PBD’s (Table 3, Q1). This inconvenience was associated with perceived time restraints in preparing plant-based meals, as well as a lack of restaurant or supermarket options available (Table 3, Q4). Therefore, this inconvenience led participants to express a lack of control over following a PBD (Table 3, Q5).

Lack of inclusiveness and accessibility: the lack of inclusiveness for people of lower socioeconomic status (SES) toward adopting a PBD was highlighted by all participants (Table 3, Q2). More specifically, PBD’s were perceived to be more expensive to adopt due to the perceived cost effectiveness of animal-based food items (Table 3, Q5, Q4). Therefore, people with less financial freedom were perceived to be less likely to follow a PBD due to financial barriers.

## Discussion

The aim of this study was to investigate the factors that may influence young people’s intentions to adopt a PBD, through using the TPB as a framework for organising the factors. The study also sought to identify the barriers and facilitators that affect young people adopting a more PBD. The findings showed that young people held mainly positive views toward adopting PBD’s. However, their intentions to follow PBD’s were influenced by several psychosocial factors, as highlighted by the TPB. Within attitudes, six sub-themes were identified: awareness of a healthy diet, environmental concerns, health concerns and distrust, confusion with veganism and stigma, perceived restriction and lack of enjoyment, and need for education. Within subjective norms, three sub-themes were identified: cultural and familial norms, peer influence, and exposure through social media. Within PBC, four sub-themes were identified: lack of independence and parental control, lack of knowledge and perceived difficulty, inconvenience, and lack of inclusiveness and accessibility. These findings provide support for the TPB in highlighting the barriers and facilitators that affect young people’s attitudes and intentions (Rosenfeld and Burrow, 2018) to follow a PBD.

### Attitudes

The findings of the current study reveal that young people held both positive and negative attitudes that can influence their intentions to follow a more PBD. Young people in the current study discussed an awareness of the importance of eating a healthy diet demonstrating their understanding of the benefits of consuming plant-based food items. This coincides with previous research which found that the uptake of PBD’s were facilitated by peoples understanding of the associated health benefits (Miki et al., 2020). Therefore, young people may be more likely to adopt a PBD through understanding the associated health benefits. This finding relates to the young people’s view that there is a need for education in helping to facilitate the likelihood of young people adopting a PBD. Education during childhood is necessary for implementing healthier eating habits into later years (Chaudhary et al., 2020). This has been evidenced by a systematic review and meta-analysis which showed that educational interventions, such as experiential learning and nutrition education programmes, promoted significant positive changes in primary school children’s knowledge and behaviors surrounding fruit and vegetable consumption (Dudley et al., 2015). Therefore, increasing education on PBD’s within schools may help to facilitate young people to transition toward a more PBD.

Young people’s positive attitudes to the associated health benefits and willingness to adopt a PBD could also be attributed to the perceived environmental benefits of reducing meat consumption. A previous study exploring attitudes between food, the environment and climate change found that Scottish adults were predominantly unwilling to reduce their meat consumption and demonstrated a lack of awareness of the link between meat consumption and the environment (Macdiarmid et al., 2016). In contrast, our own findings indicate a willingness of the young people sampled to adopt a PBD with many being influenced by the perceived environmental benefits of reducing meat consumption. This finding reflects research that found that environmental concerns predicted the consumption of vegetarian diets (Fox and Ward, 2008) and for adopting PBDs (Rosenfeld, 2019).

Despite knowledge of the value of a healthy diet and the role of nutrient-rich plant-based foods, young people also expressed some health concerns toward consuming PBD’s, which reflects research that found that Australian adults were reluctant to reduce their meat consumption due to health concerns of lacking protein and dietary nutrients normally gained from meat (Bogueva et al., 2017). This negative attitude was highlighted through health concerns focusing on the importance of meat for a key protein source to provide strength. This concern was predominantly observed in male participants supporting previous findings that indicate that males exhibited greater associations between meat and strength than females (Love and Sulikowski, 2018). In contrast, some females in the study expressed concerns toward people developing eating disorders through restricting animal-based items. Vegan and vegetarian diets can facilitate dysfunctional eating due to their restrictive nature (Fuller et al., 2022), which may explain the female participants concerns highlighted in the present study.

Further negative attitudinal barriers held by young people which should be considered as influencing their intention to follow PBDs included a perceived restriction and lack of enjoyment. Research has demonstrated that across European countries, young adults who perceived PBD’s as tasty were more likely to follow a PBD (Faber et al., 2020). An explanation for this may be provided by Kahneman and Tversky’s (1979) prospect theory, which posits that individuals evaluate gains and losses differently, but are more loss aversive to certain goods. Many young people in the current study may have perceived PBD’s to be loss-framed rather than gain-framed, since veganism is defined through what it lacks (Kent et al., 2022), and the participants confused PBD’s with vegan diets. Young people may perceive PBD’s as a loss rather than a gain of favorable food items and thus may act as a barrier to adopting a PBD.

In the current study, there was often a perception of PBD’s with veganism and negative stereotyping of PBD’s, which supports research by De Groeve et al. (2021), who found that non-meat diets were more negatively stereotyped and viewed as less socially attractive. People who consume meat-based diets may hold ambivalent views toward non-meat eaters (Willett et al., 2019). Most notably, these people may hold positive views toward non-meat eaters due to their moral commitment toward helping the environment but may also feel threatened by non-meat eater’s moral identity. Thus, people will defend their own dietary behaviors by negatively stereotyping non-meat eaters as being overcommitted (Willett et al., 2019). This was termed the ‘meat paradox’, which is believed to emerge from the cognitive dissonance that meat eaters may experience (Loughnan et al., 2014). Therefore, this attitude may have negatively impacted on the participants intentions to follow a PBD due to the misconception of PBD’s with vegan stereotypes.

### Subjective norms

The perceived cultural and familial norms surrounding meat consumption was also a barrier toward following PBD’s. It has been argued that young people’s adherence to the traditional cultural norms of eating meat in the United Kingdom may reinforce their national social identity and make it difficult to change dietary habits (Nguyen and Platow, 2021). Similarly, British adults were shown to be less likely to follow PBD’s when they perceived significant others to disapprove of consuming PBD’s (Sharps et al., 2021). Thus, social identity and significant others can play an important role in influencing or reinforcing consumption behavior.

In contrast to the previous barrier of cultural and familial norms, peer influence was found to facilitate young people’s perceived acceptability of following PBD’s. Research has shown that people in closer social proximity to others, including students attending university, influenced dietary behaviors more than people in distant social groups (Cruwys et al., 2015). Further, the phenomenon of social modeling, whereby individuals modify their dietary intake to match that of their peers, may positively influence dietary behaviors (Cruwys et al., 2015). Peers can aid exposure to new food items available and encourage others to adopt similar diets to themselves (Ge et al., 2022). The role of social media may further facilitate young people’s likelihood of following PBD’s. Studies have shown that greater exposure to foods increases their acceptance and likability (e.g., Fildes et al., 2014), which reflects research that demonstrated that social media exposure to food messaging was positively associated with young peoples perceived norms toward food (Qutteina et al., 2022). Therefore, exposure to PBD’s through social media may encourage young people to follow PBD’s.

### Perceived behavioral control

The misconception of PBD’s with veganism found in the present study, appeared to be linked to the barriers of perceived difficulty and lack of knowledge toward PBD’s, which supports research by Vanhonacker et al. (2013). Young people often lack an understanding of how to easily prepare PBD’s. The perceived inconvenience of following a PBD, particularly regarding the preparation and cooking of plant-based meals, reflects research by Fehér et al. (2020), who found that Hungarian participants perceived PBD’s to be time consuming to prepare. Therefore, the perceived difficulty, lack of knowledge, and inconvenience in meal preparation are important barriers toward young people’s adoption of PBD’s and therefore education can play an important role in helping to overcome this. Furthermore, the perceived lack of accessibility and inclusiveness expressed in the current study highlights a perceived financial barrier to following a PBD. Plant-based food items were often noted as expensive and not always easily accessible. Furthermore, young people may be experiencing financial constraints and independence living away from primary caretakers (Moreno et al., 2014), and the perceived cost can prevent the uptake of vegan, vegetarian and PBD’s (Povey et al., 2001; Lea et al., 2006). Thus, cost and financial constraints are important barriers that may prevent young people from following a PBD.

Many young people undergo a stage of financial independence with moving away from the home which may impact on their dietary and food purchasing options, however for other young people a lack of independence around meal choices as they continue living at home with parental control of food purchasing and preparation could negatively influence their ability to follow PBD’s. Parents have a central role in young people’s food choices (Scaglioni et al., 2008), and research has demonstrated that increased parental control is associated with reduced vegetable consumption in children (Fisher et al., 2002). This can be attributed to parents choosing and preparing foods for the household to eat (Savage et al., 2007) which may be linked to the family’s norms and attitudes to diet. Although young people experience a developmental period with greater independence, parents may continue to influence dietary choices, particularly when meals are prepared within the family environment (Giskes et al., 2005). Therefore, young people who lack independence and experience greater parental control may have less opportunity and thus be less likely to consume a PBD.

### Implications of research

This study highlights important implications for public health promotion practice and policy for PBDs. By understanding young people’s views toward PBD’s, health promotion strategies can be tailored to motivate young people to consume a more PBD. This may be achieved through encouraging healthcare professionals’ and policy makers to effectively communicate the positive health outcomes of reducing meat intake. Greater communication can help change young people’s attitudes toward the positive and negative health outcomes of following a PBD (Wolstenholme et al., 2021).

Health promotion strategies should also consider the role of subjective norms surrounding PBD’s. This can be achieved through framing health messages to portray following PBD’s as normative behavior, which may motivate young people to reduce their meat consumption (Wolstenholme et al., 2021). Additionally, interventions that target reducing meat consumption should aim to increase people’s PBC by highlighting alternative food options and promoting convenient recipes for young people. This can help increase young people’s perceived ability to follow a PBD.

### Strengths, limitations, and future directions

This study has contributed evidence to a small body of literature on young people’s thoughts and attitudes toward PBD’s in the United Kingdom. By employing a qualitative research methodology, this study has provided a richer understanding of young people’s views toward PBD’s. Additionally, using the TPB as a framework allowed for a focused and structured understanding of behavior, attitudes, and intentions toward PBD’s.

Despite this study’s strengths, there are several limitations that should be addressed. Firstly, most participants recruited identified as female (76%). This may have reduced the generalisability of the findings, as research has demonstrated that food choices can differ between genders (Manippa et al., 2017). Further, research has found that women may be more likely to perceive meat as unhealthier compared with males (Bärebring et al., 2020). Therefore, future research should recruit an equal gender demographic to enhance the validity of findings on young people’s dietary attitudes. A second limitation may be due to all the participants being students enrolled in higher education. Our study sought to investigate the views of young people from the general population, hence recruitment was conducted using social media advertisement. Nevertheless, participants who volunteered for this study were enrolled in higher education. Students typically have higher SES than non-students, which can reduce the applicability of results (Hanel and Vione, 2016) and thus may impact the generalisability of this study’s findings (Saucier et al., 2015). Therefore, future research should aim to recruit participants from varying socio-economic demographics to further understand the dietary behaviors of young people who are not enrolled in further education. Lastly, although qualitative research provides a richer understanding of phenomena, researchers have argued that it may be inevitably subjective (Schraube and Højholt, 2019). Triangulation, whereby qualitative and quantitative approaches are simultaneously employed, may enhance the credibility of findings (Restivo and Apostolidis, 2019). Triangulation has been shown to reduce biasing results through directly comparing qualitative findings with quantitative findings, which may better explain phenomenon (Restivo and Apostolidis, 2019). Therefore, future research should employ triangulation to further understand attitudes toward PBD’s.

Overall, this study provides a valuable insight into young people’s views of PBD’s in the United Kingdom. More specifically, our use of the TPB as a framework identified that young people’s attitudes were influenced by an awareness of a healthy diet, perceived environmental concerns, perceptions of PBD’s with negative stereotypes, perceived restriction and lack of enjoyment, distrust toward health benefits, and need for education. Subjective norms were guided by cultural and familial norms, peer influence, and social media. Additionally, participants PBC was influenced by a lack of independence and parental control, a lack of knowledge and perceived difficulty, a lack of inclusiveness and accessibility, and inconvenience in engaging with a PBD. These findings support previous research conducted in other western countries contributing evidence on young people’s views toward PBD’s and highlight the need for more education and widening access to increase young people’s knowledge and willingness to consume PBD’s.

## Data availability statement

The datasets presented in this article are not readily available because of data sharing conditions in the participant consent. Requests to access the datasets should be directed to the corresponding author.

## Ethics statement

The studies involving humans were approved by the University of St Andrews Medicine Ethics committee. The studies were conducted in accordance with the local legislation and institutional requirements. The participants provided their written informed consent to participate in this study.

## Author contributions

CM, SC, and JC have made a substantial, direct, and intellectual contribution to the design and development of the work, analysis of the data, editing of the work, and approval of the final version to be published.

## Funding

This work was supported by the University of St Andrews.

## Conflict of interest

The authors declare that the research was conducted in the absence of any commercial or financial relationships that could be construed as a potential conflict of interest.

## Publisher’s note

All claims expressed in this article are solely those of the authors and do not necessarily represent those of their affiliated organizations, or those of the publisher, the editors and the reviewers. Any product that may be evaluated in this article, or claim that may be made by its manufacturer, is not guaranteed or endorsed by the publisher.
