# Supplementary material for: A qualitative study of young peoples’ thoughts and attitudes to follow a more plant-based diet
Source: Front Psychol. 2023 Aug 31;14:1196142. doi: 10.3389/fpsyg.2023.1196142 (PMC10506079; doi:10.3389/fpsyg.2023.1196142)
Supplement: Supplementary file 1 [file Table_1.DOCX]

**Interview Guide**

1. What age are you?
2. What ethnicity do you identify as?
3. What gender do you identify with?
4. Can you describe your highest level of education (Highschool, University, Postgraduate?)
5. Can you describe how concerned/unconcerned you are about the environment?
6. Are you/have you been involved with any environmentally friendly activities or organisations before? (Volunteering or day to day things like recycling).

***Prompt:*** *If yes, can you explain these further?*

***Prompt:*** *Why? Why not is this important to you?*

1. Can you describe an example of what your current diet consists of in a day (Breakfast to dinner)?

***Prompt:*** *Have you always followed a similar diet throughout your lifetime? when and how has it differed?*

1. Do you think it is important to maintain a well-balanced diet? If so, why?
2. Have you heard of the term plant-based diets?

***Prompt*** *If yes, do you know what a plant-based diet consists of? what do you think a plant-based diet consists of?*

***Prompt*** *If no/unsure, plant-based diets consist of less animal-based products, such as dairy, and incorporate more nutrient dense food items such as fruits, vegetables, nuts, beans, lentils, and seeds.*

1. Do you currently eat any plant-based items, and if so, can you name these foods/meals?

***Prompt*** *If can name, would you say you enjoy/dislike the taste of plant-based foods?*

1. What are your current views surrounding a plant-based diet?

***Prompt*** *Have you ever considered adopting a more plant-based diet?*

***Prompt*** *Can you describe how motivated/unmotivated you would be to adopt a plant-based diet, and why?*

1. Are there any benefits you believe to the environment by adopting a more plant-based diet?

***Prompt*** *If yes, what are these?*

***Prompt*** *If no, can you explain why?*

1. Are there any benefits you believe to people’s health by adopting a more plant-based diet? What are these?

***Prompt:*** *If no, why do you think this?*

1. Are there any disbenefits (disadvantages) or risks to peoples’ health or the environment by adopting a plant-based diet?

***Prompt:*** *What are these?*

1. Are there any barriers or facilitators to yourself and the wider population you believe to people adopting a plant-based diet? What are these?
2. What are your views on plant-based foods becoming alternative food choices for animal-based products (such as oat milk or meat free items like Quorn)?
3. Do you think there should be more education (in schools, or advertisements) on plant-based-diets?
4. Do you know anyone who currently follows a plant-based diet?

***Prompt:*** *If ‘yes’, do you think it has affected your dietary choices?*

1. Is there anything that currently influences your choices in food (health reasons, taste, friends, friendship groups, etc)? Can you explain further?
2. Do you find it important what your friends or family think about what you eat?

***Prompt:****” If ‘yes’, why do you believe it important what they think?*

1. Has social media or external people influenced your dietary choices previously or currently?

***Prompt:*** *If ‘yes’, can you explain further.*

1. Are there any perceived stereotypes to following a plant-based diet?

***Prompt:*** *if ‘yes’, can you describe these stereotypes?*

1. Can you describe whether you make a lot of your meals by yourself or do other people cook for you?
2. Do you think you would be able to make meals consisting of plant-based alternative food items?

***Prompt:*** *If ‘yes’, can you state how easy/difficult you believe this would be? Do you think you would be able to stick to this for an extended period of time?*

1. Can you think of any ideas that might help people become more plant-based?

***Prompt:*** *If ‘yes’ can you explain them further?*

1. What do you think encourages or prevents you and the people around you from following a more plant-based diet?
2. Are there any questions you would like to ask about this interview, or thoughts you wish to share?
